# Shifting pattern of gut microbiota in pregnant women two decades apart – an observational study

**DOI:** 10.1080/19490976.2023.2234656

**Published:** 2023-07-19

**Authors:** Samuli Rautava, Marta Selma-Royo, Teo Oksanen, Maria Carmen Collado, Erika Isolauri

**Affiliations:** a Department of Clinical Sciences, Faculty of Medicine, University of Turku, Turku, Finland; b Department of Pediatrics, University of Helsinki and New Children’s Hospital, Helsinki University Hospital, Helsinki, Finland; c Institute of Agrochemistry and Food Technology-National Research Council (IATA- CSIC), Valencia, Spain; d Department of Paediatrics and Adolescent Medicine, Turku University Hospital, Turku, Finland

**Keywords:** Fecal short-chain fatty acids, gut microbiota, obesity, overweight, pregnancy

## Abstract

**Background:**

Past decades have witnessed a decrease in environmental biodiversity. We hypothesized a similar decrease in indigenous gut microbiota diversity, which may have contributed to the obesity epidemic.

**Objective:**

To investigate the changes in the composition and function of the gut microbiota in pregnant women over a period of 20 years.

**Study design:**

Altogether 124 pregnant women (41 overweight and matched 83 normal weight) pregnant in 1997, 2007 or 2017 were included in the study. The gut microbiota composition was assessed from fecal samples obtained at 32 weeks of gestation by 16S rRNA gene sequencing. Fecal short chain fatty acid (SCFA) profiles were measured by gas chromatography mass spectrometry (GC-MS).

**Results:**

Distinct gut microbiota profiles were detected in pregnant women from 1997, 2007 and 2017 (PERMANOVA Bray-Curtis R^2^ = 0.029, *p* = 0.001). The women pregnant in 1997 exhibited significantly higher microbiota richness and diversity as compared to those pregnant in 2007 and 2017. The total concentration of fecal SCFAs was significantly higher in the pregnant women in 1997 compared to those in 2007 and 2017. Significant differences in gut microbiota composition between normal weight and overweight women were manifest in 1997 but not in 2007 or 2017.

**Conclusions:**

The decrease in intestinal microbiota richness and diversity over two decades occurred in parallel with the decline in biodiversity in our natural surroundings. It appears that the gut microbiota of pregnant women has changed over time to a composition typical for overweight individuals.

## Plain Language Summary

The composition of the indigenous gut microbiota was investigated in pregnant women from three different time periods (1997, 2007 and 2017) in the same geographical and cultural area in Southwest Finland. Distinct gut microbiota profiles were evident in the women from the different time periods. The women pregnant in 1997 exhibited significantly higher microbiota richness and diversity as compared to the pregnant women from 2007 to 2017. The cause of the loss of gut microbiota richness and diversity over time remains obscure, since no major changes in the population, dietary practices or antibiotic use occurred in the area during the course of the study periods. Gut microbiota composition has been suggested to play a causal role in the development of overweight and obesity. In line with this notion, significant differences in the gut microbiota composition between normal weight and overweight were detectable in women pregnant in 1997. However, no such differences were manifest in women pregnant in 2007 or 2017 and the gut microbiota of these individuals resembled that of overweight pregnant women from 1997. The results of the study provide direct evidence for a decline in gut microbiota diversity over time in the same geographical area and the same population. It furthermore appears that the gut microbiota of pregnant women has changed over time to a composition typical for overweight individuals. The gut microbiota profiles may thus provide insight into the development and intergenerational transfer of overweight.

## Introduction

The past decades have witnessed a shift in human morbidity characterized by the rise of non-communicable diseases with phenotypes ranging from allergic, autoimmune, and inflammatory diseases to obesity.^1^ The World Health Organization recognized obesity as a global epidemic in 1997.^2^ Acceleration of the obesity epidemic with comorbidities is expected in the future since excessive weight gain is prevalent in children^3^ and in the population at reproductive age.^4^ The development of overweight and obesity in childhood is multifactorial but it is well established that maternal obesity markedly increases the risk for childhood obesity.^5^ Given that obesity in children is likely to persist into adulthood, a vicious cycle of intergenerational obesity development is likely to arise.^6^ The primary cause of overweight and obesity is excessive intake of energy compared to expenditure. The obesity epidemic may to a large extent be attributed to changes in diet and a shift to a more sedentary lifestyle over the past decades. Interestingly, changes in the composition and function of the gut microbiota have also been linked to obesity.^7^ Intestinal microecology changes in response to an unhealthy diet, but aberrant gut microbiota composition has been reported already during infancy before the onset of overweight.^8^ In addition, experimental studies conducted using animal models suggest a causal role for the gut microbiota in the development of overweight and obesity through increased dietary energy harvest, changes in satiety and modulation of host metabolism.^9^ The gut microbiota may propagate inflammatory responses recognized to underlie overweight and obesity but may also act as a source of microbial stimuli which attenuate the chronic low-grade systemic inflammation.^8,10–13^

The hypothesized causal link between the gut microbiota and obesity development may in part be mediated through the production of metabolites such as short-chain fatty acids (SCFA), which have multiple functions including regulation of energy metabolism and immune function and stabilization of gut barrier function.^9^ In line with this, low fecal concentrations of SCFAs have been detected in overweight as compared to normal weight children.^14^ Early interaction with microbial antigens and the establishment of an age and environment-appropriate gut microbiota is thus anticipated to ensure normal growth and healthy development.^8,13,15^ The present study builds upon a unifying theory on these two parallel phenomena: the global increase in the prevalence of overweight and obesity,^2^ and the potential causal role of the gut microbiota in the obese state.^16^ To achieve a comprehensive characterization of the evolution of the gut microbiota in pregnant women over the course of the obesity epidemic, we investigated the gut microbiota composition and function in the third trimester of pregnancy in individuals with normal or high pre-pregnancy body mass index (BMI) in 1997, 2007 and 2017.

## Results

**Figure 1.**
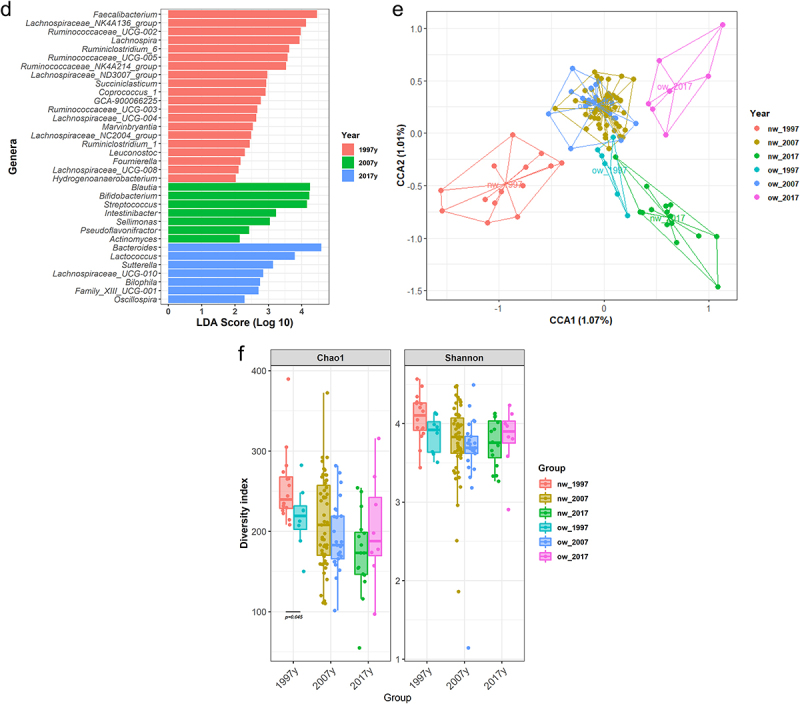
(Continued).

Altogether 124 pregnant women (24 pregnant in 1997, 76 in 2007 and 24 in 2017) were included in the study. Pre-pregnancy overweight was present in 41 subjects whilst 83 women were normal weight (Supplementary Figure S1). The baseline and pregnancy characteristics of the pregnant women are presented in Table 1. The time period (1997, 2007 or 2017) followed by pre-pregnancy BMI group (pre-pregnancy BMI 18.5–24.9 or ≥25 kg/m^2^) and gestational weight gain were the most relevant factors contributing to the gut microbiota composition of pregnant women according to multivariate analysis of the microbial beta diversity based on the Bray-Curtis distance (Supplementary Figure S2a). When all study subjects were included in the analysis, statistically significant differences were detected in the gut microbiota profiles of pregnant women among the three time periods (permutational multivariate analysis of variance (PERMANOVA) Bray-Curtis R2 = 0.029, *p* = 0.001) (Figure 1a). The observed differences were confirmed by multivariate canonical correspondence analysis (CCA) (F = 1.17, chi square = 0.34, *p* = 0.002; Figure 1b). The gut microbiota differences were reflected in alpha-diversity, as the pregnant women from 1997 exhibited higher microbial richness and diversity as measured by the Chao1 and Shannon indices, respectively, as compared to the pregnant women from 2007 to 2017. In general, the microbial richness was significantly reduced over time from 1997 to 2017 (Figure 1c).
**Figure 1.**
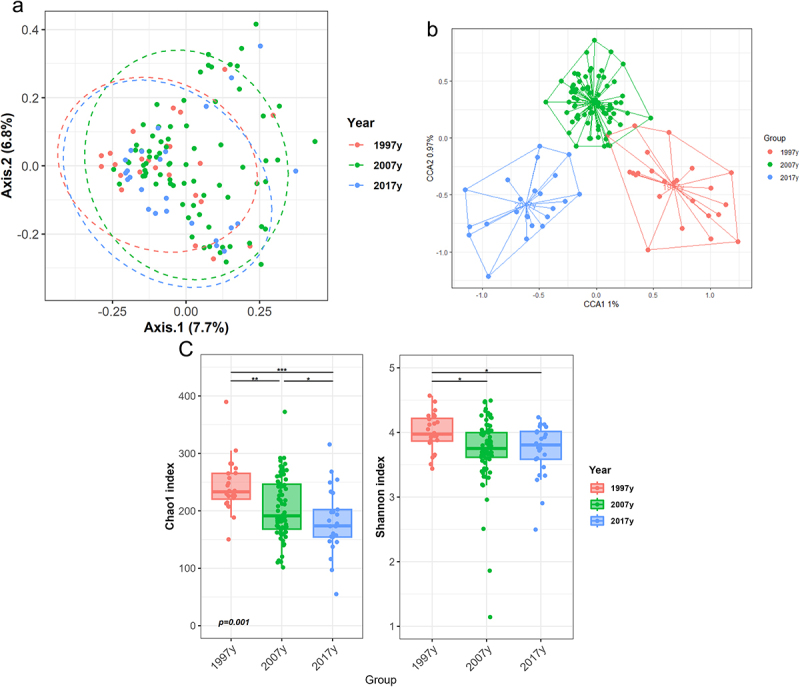
Composition and diversity of the gut microbiota in normal weight and overweight pregnant women over a period of two decades.

(a) Principal coordinate analysis (PCA) of the gut microbiota composition at genus level from pregnant women from three time periods (1997, 2007 and 2017). The effect of the time period on microbiota composition reported by canonical correspondence analysis (CCA) (b) and differences in alpha-diversity (Shannon index) and richness (Chao1 index) at different time periods (c). Linear discriminant analysis (LDA) effect size (LEfSe) analysis of gut microbiota differences along the time periods (d). The bar graph presents the LDA scores marking the taxa enriched in each time period. The link with time period and maternal pregestational body mass index (BMI) and the gut microbiota as assessed by CCA (e) and the alpha-diversity indexes (Shannon index) and richness (Chao1 index) (f). Statistical testing was performed by the Kruskal–Wallis test followed by post-hoc Dunn’s test with Benjamini–Hochberg adjustment. * *p* < .05, ** *p* < .01, *** *p* < .001.

**Table 1.** Clinical characteristics of the pregnant women in 1997, 2007 and 2017.

	1997			2007			2017					
	Total	NW	OW	Total	NW	OW	Total	NW	OW	Total	P-value NW	P-value OW
Number of women	24	16	8	76	51	25	24	16	8			
Age (y), mean (SD)	29 (4.07)	27.5 (3.43)	32.0 (3.75)	30.7 (3.86)	30.3 (3.77)	31.7 (3.97)	32.8 (2.93)	32.8 (2.98)	33.0 (3.03)	**0.003**	<.001	.699
Pre-pregnancy BMI (kg/m^2^, median (IQR)^¥^	20.80 (20.4–25.2)	20.7 (20.2–20.8)	25.6 (25.1–26.6)	21.50 (20.3–26.7)	20.6 (20.1–21.5)	28.6 (26.7–31.2)	22.3 (20.5–25.6)	21.2 (19.9–22.6)	25.9 (25.5–28.4)	0.539	.367	.004
Weight gain during pregnancy (kg), median (IQR)	14.25 (9.3–16.2)	12.5 (9.1–15.6)	16.1 (12.4–20.3)	13.50 (11.8–16.0)	13.8 (12.2–16.0)	11.9 (8.8–15.7)	14.85 (10.4–16.5)	14.9 (12.3–16.5)	13.4 (7.8–17.3)	0.945	.352	.307
Gestational age at delivery (wk), median (IQR)	39.5 (39–40)	40 (38.3–40.0)	39 (39–40)	40 (39–41)	40.0 (39–41.0)	40.0 (38.5–41.0)	39 (38–39)	39.0 (37.7–40.0)	39 (37.3–39.0)	**0.002**	.010	.174
Number of previous pregnancies, mean (IQR)	1 (1–2)	1 (1–2)	2 (1–2.8)	1 (1–2.8)	1 (1–2)	2 (1–3)	2 (1–3)	2 (1–3.8)	2.5 (2–3)	**0.038**	.136	.375
Parity, median (IQR)	0 (0–0.8)	0 (0–0)	0 (0–1)	0 (0–1)	0 (0–1)	1.0 (0–2)	1 (0–1)	0 (0–1)	1 (1–1.8)	0.050	.353	.072
Antibiotics during pregnancy, yes/no	6/18	5/11	1/7	12/64	6/45	6/19	6/18	5/11	1/7	0.450	.091	.663

¥ Pre-pregnancy BMI between 18.5–24.9 kg/m^2^ was considered to represent normal weight (NW), while BMI greater or equal to 25 kg/m^2^ was considered to represent overweight (OW). Women with obesity (BMI greater or equal to 30 kg/m^2^) were classified in the same category as those with overweight.

Distribution of the variables was assessed by Shapiro–Wilk test. Differences in categories of normally and non-normally distributed variables were calculated by ANOVA test or Kruskal–Wallis test, respectively. Chi-square test were used to assess differences in antibiotic intake during pregnancy between categories. p-values <.05 were considered statistically significant. SD: Standard deviation. IQR: Interquartile range.

The phylum Firmicutes was enriched in the microbiota of pregnant women from 1997 (*p* < .001, q=<.001) compared to women from 2007 to 2017. At genus level (Supplementary Figure S2b), the main differences were characterized by the higher abundance of several groups from the families *Lachnospiraceae* and *Ruminococaccaceae* such as *Lachnospira* genus (*p* = 0.002, q = 0.043), *Lachnospiraceae* NK4A136 group (*p* = 0.021, q = 0.19), *Lachnospiraceae*_UCG-8 (*p* = <.001, q = 0.022), UCG-10 (*p* = 0.004, q = 0.059), *Faecalibacterium* (*p* = 0.020, q = 0.190), *Ruminococacceae* UCG-002 (*p* = 0.002, q = 0.044) or unclassified *Ruminococcaceae* genera (*p* < .001, q = 0.022) in the gut microbiota of pregnant women from 1997 compared with those from 2007 to 2017. In addition, the gut microbiota of women pregnant in 1997 was characterized by high relative abundance of *Faecalibacterium* and groups from *Lachnospiraceae* and *Ruminococcaceae* (most of them short-chain fatty acid (SCFA) producers), while in women pregnant in 2007 *Streptococcus* and *Bifidobacterium* were the predominant genera as assessed by LEfSe (Figure 1d). In 2017, the gut microbiota of pregnant women was characterized by the genera *Bacteroides* and *Bilophila*.

We next investigated the gut microbiota composition in the subgroups of normal weight and overweight pregnant women over the course of the study periods. A multivariate Maaslin analysis considering the study period and BMI group as factors revealed that the phylum Bacteroidetes (*p* = 0.005, q = 0.074), mainly due the genus *Bacteroides* (*p* = 0.008, q = 0.146), was more abundant in normal weight compared to overweight pregnant women (Supplementary Table S1). Multivariate CCA revealed also significant differences in the gut microbiota composition of pregnant women among the three time periods dependent on pre-pregnancy BMI (F = 1.06, chi square = 0.80, *p* = 0.004, Figure 1e). A clear distinction in the gut microbiota composition between normal weight and overweight women was manifest only in the 1997 population. Decreased gut microbiota richness was observed in overweight compared to normal weight women (*p* = 0.045) in 1997 (Figure 1f) while no such difference was observed in 2007 or 2017.

**Figure 2.**
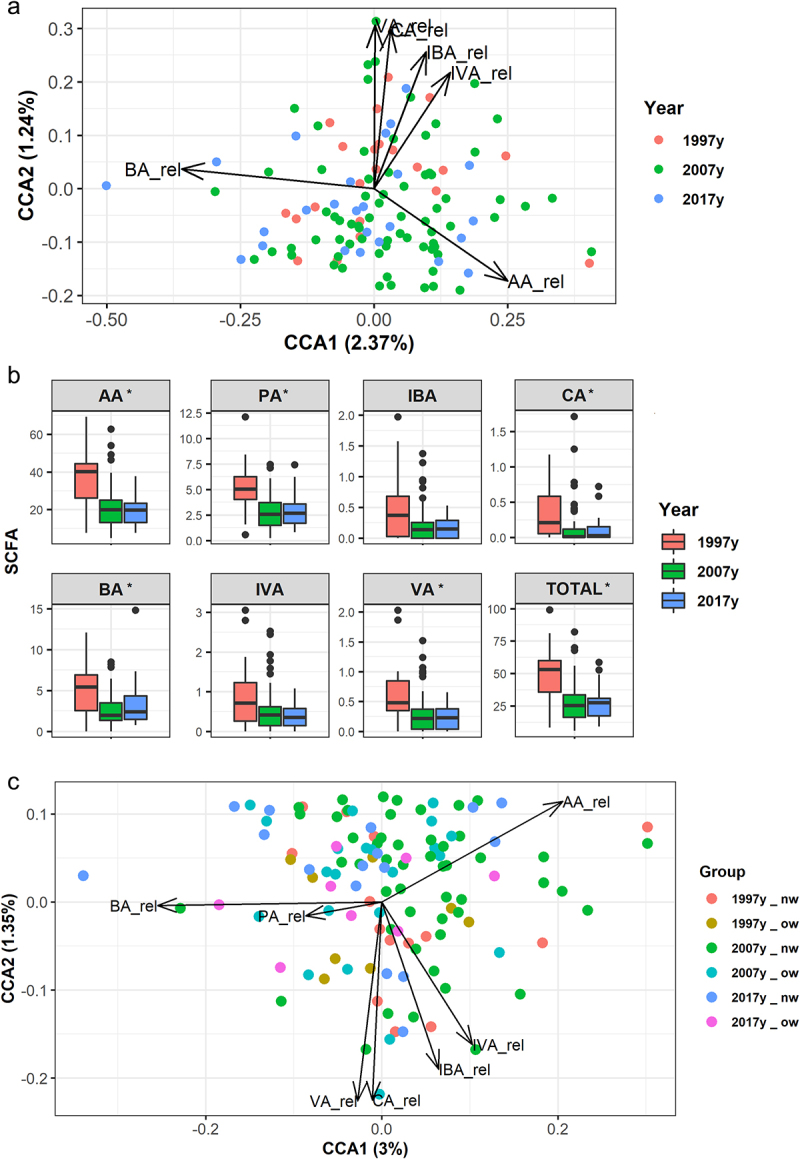
Composition, diversity and activity of the gut microbiota in pregnant women over a period of two decades.

Differences in gut microbiota activity as measured by fecal SCFA concentrations were observed among the whole population of pregnant women from different time periods (PERMANOVA Euclidean distances R2 = 0.185, *p* = 0.001). The differences were also confirmed by multivariate CCA (F = 2.19, chi square = 0.002, *p* = 0.037; Figure 2a). The total concentration of SCFAs was significantly higher (*p* < .001) in the pregnant women in 1997 compared to those in 2007 and 2017, mainly due to higher levels of acetate (*p* < .001) and butyrate (*p* < .001). When the relative abundances of each individual SCFA were corrected by the total SCFA production, the women pregnant in 1997 exhibited a trend for higher production of some minor metabolites such as caproic acid (*p* = 0.063) and valeric acid (*p* = 0.053) (Figure 2b). Higher levels of SCFA were associated with higher abundance of *Ruminococcaceae* and *Lachnospiraceae members* (Supplementary Figure S3). Genera from these families showed a positive relation with a rho ranging 0.2–0.4 especially with minor SCFAs (IVA, VA and CA) while other genera such as *Veillonella* or *Haemophilus* were negatively correlated with those SCFA.

(a) Canonical Correspondence (CCA) depicting the organization of the fecal short-chain fatty acid (SCFA) profiles in the time periods and the association of each time period with the presence of each SCFA individually. (b) Boxplots of the differences in each SCFA quantification in the fecal samples according to time period. Statistical analysis was performed using Kruskal–Wallis test between groups. (c) CCA showing the association of both pre-gestational BMI and time period in the fecal SCFA profiles.

CCA models considering time period and BMI groups showed distinct fecal SCFA profiles (F = 1.905, chi square = 0.0014, *p* = 0.029; Figure 2c) with the time period emerging as the most significant factor (*p* = 0.025). Despite the higher levels of SCFA in 1997 and a significant decrease at later time periods, no significant differences were observed either in total SCFA levels or the concentrations of acetate, butyrate, and the rest of SCFAs between normal weight and overweight women.

## Discussion

A decrease in intestinal microbiota richness and diversity over time was observed in the pregnant women investigated in 1997, 2007 and 2017. In addition, the fecal concentration of SCFAs, an indicator of gut microbiota function, was significantly higher in the women pregnant in 1997 as compared to the later time points. In the subjects pregnant in 1997, distinct clustering of the gut microbiota composition was evident between normal weight and overweight women while no such differences were detected in 2007 or 2017. The gut microbiota of overweight and normal weight women in 2007 resembled the gut microbiota of overweight women from 1997. The gut microbiota of pregnant women may thus have changed to a composition typical for overweight individuals.

The continuous global decline in biodiversity is a major threat facing modern humanity, but we are only beginning to understand its consequences on human health. Our direct interaction with nature is diminishing with the proliferation of the industrialized and urbanized lifestyle.^17^ The changes in our external environment, resulting from the agricultural revolution, population growth and urbanization have begun millennia ago, but the velocity of lifestyle modernization and biodiversity decline have been unprecedently rapid over the past decades. Our results suggest that a parallel decline in microbial diversity has occurred in our internal environment during the last decades. Whether there is a causal link between reduced biodiversity in our external environment and the gut microbiota remains an open question, but there are data indicating that environmental exposures represent the driving force behind the differences in gut microbiota composition between individuals.^18^

The emergence of non-communicable disease (NCD) and obesity may be explained by reduced microbial contact resulting from changes in hygiene, medical practices, diet, living conditions and lifestyle.^12^ However, we lack direct evidence for the external microbial environment modulating the indigenous microbiota. The pregnant women investigated in the present study were all living in the same geographical area, with no obvious changes in population density or natural surroundings during the study period. The specific exposures underlying the observed compositional differences thus remain unknown. Previously, antibiotic use has been linked with both gut microbiota perturbations and increased prevalence of overweight and obesity.^8,19^ Interestingly, however, the use of all major groups of antibiotics has declined in Finland during recent years according to the Finnish Institute for Health and Welfare (https://urn.fi/URN:ISBN:978-952-343-425-7). Data on antibiotic use before pregnancy by the women in the present study were unfortunately not available. The number of women exposed to antibiotics during pregnancy was similar among the time periods (Table 1). Diet is the most substantial modulator of the gut microbiota^20^ and changes in dietary practices may explain our results. According to a national longitudinal survey with data from the years 1997, 2007 and 2017, the intake of carbohydrates and particularly sucrose decreased in women in Finland while the intakes of fiber, protein, and fat increased.^21^ The consumption of red or processed meat, sugar sweetened drinks or candy remained at the same level. Previously published data from the present population indicate similar trends of increased protein and fiber intake but no changes in the amount or quality of consumed fat in pregnant women.^22–24^ The differences compared to non-pregnant women may be explained by the fact that the pregnant women received dietary guidance at prenatal visits and are likely to pay more attention to healthy eating habits.^23,24^

The changes observed in the gut microbiota composition and activity in pregnant women may have clinical significance. We have previously reported that the gut microbiota undergoes marked changes during the course of pregnancy along with the physiological weight gain and insulin resistance necessary for fetal growth.^25^ Reduced diversity and potential to induce adiposity and reduced insulin sensitivity in experimental animals was detected in the gut microbiota during the third trimester in comparison to early pregnancy.^25^ According to a recent report, the gut microbiota of women with pre-pregnancy overweight or obesity is characterized by lower diversity and functional changes as compared to that of normal weight women.^26^ The importance of these phenomena is underscored by data indicating that the development of gestational diabetes mellitus (GDM) is preceded by changes in gut microbiota composition and reduced concentration of fecal SCFAs.^27^ Fecal microbiota transplant using samples obtained during early pregnancy from women who later developed GDM to germ-free mice resulted in insulin resistance, which may be interpreted to suggest a causal role for the gut microbiota in the development of GDM. The health consequences of aberrant maternal gut microbiota may extend to the next generation. The mother provides the first and most important inoculum in the microbial colonization of the child and early infancy gut microbiota changes and reduced fecal SCFAs have been associated with obesity development.^8,14^ The altered maternal gut microbiota composition and function, including reduced production of SCFAs, may thus contribute to the intergenerational transfer of obesity as the aberrant microbiota and, consequently, the seeds for obesity development, are passed on to the offspring by different routes: during pregnancy, at delivery by the microbes in the birth canal, and close contact with the mother during breastfeeding.

Some women in our study had remained normal weight despite their microbiota resembling that of those who are overweight. This suggests that some individuals may harbor resilience factors protecting against the development of adverse outcomes. The increasing prevalence of chronic inflammatory disease and obesity have spawn interest in identifying microbial factors that could support healthier immune and metabolic development and reduce disease risk.^28^ The present study calls for more clinical research conceptualizing how the human microbiota is linked to disease risk, and specifically, a search for a microbiota profile as an indicator of the risk of adverse outcome in the child. These data would provide a basis for personalized nutrition solutions which, together with a healthy dietary intake, might benefit pregnant women and their children.

Our study has several limitations, which should be considered when interpreting the results. The number of subjects is relatively small and may prevent detecting subtle but significant differences in gut microbiota composition particularly in the subgroup analyses of normal weight and overweight women. Furthermore, the subjects were categorized to two groups based on pre-pregnancy BMI in an attempt to preserve statistical power. Categorizing individuals with overweight or obesity in the same group prevents the detection of potentially important differences between these BMI groups. As discussed above, data regarding diet or antibiotic use before pregnancy in the study subjects were not available. Even though the fecal samples were collected using a uniform protocol and the DNA extraction and sequencing were performed at the same time with the same protocol and equipment, the storage times varied considerably as per the study design, which may cause bias. However, we consider this unlikely particularly in the light of the present findings. Firstly, in a recent study, fecal and mucosal samples from a colorectal cancer biobank were stored at −80°C for a mean duration of five years. Samples stored for up to eight years were used and storage conditions had no significant influence on the results.^29^ Secondly, in the present study, no differences in DNA efficiency or concentrations were evident between the time periods and higher microbial richness and diversity was found in the older samples. Moreover, the differences between overweight and normal weight women from the same time period in 1997 could not have been caused by differences in storage time.

In parallel with the complex decline in biodiversity in our natural surroundings, a marked decrease in intestinal microbiota richness and diversity accompanied by functional changes has taken place in pregnant women over the course of two decades. The gut microbiota of pregnant women has changed toward a composition characteristic of overweight individuals. Determining the intergenerational transfer of obesity resulting from deviant initial microbiota to excessive weight gain or resilience will offer breakthroughs for key discoveries of next-generation personalized dietary interventions.

## Materials and methods

### Study participants and design

The study population consisted of pregnant women from a series of intervention studies with long-term follow-up conducted at the Turku University Hospital in Turku, Finland.^30,31^ Pregnant women recruited in 1997, 2007 and 2017 were eligible for the present study. The first inclusion criterion was availability of a fecal sample collected at 32 weeks of pregnancy prior to any microbiota-targeted interventions. The second criterion was that the information on accurate BMI was available before the pregnancy. These criteria were fulfilled by 59, 118 and 31 women in 1997, 2007 and 2017, respectively. Next, pregnant women were divided into two groups based on pre-pregnancy BMI. BMIs between 18.5 and 24.9 kg/m^2^ were considered to represent normal weight, while in overweight women BMI was greater or equal to 25 kg/m^2^. Women with obesity (BMI greater or equal to 30 kg/m^2^) were classified in the same category as those with overweight. Altogether 41 overweight women were identified (Table 1, Supplementary Figure 3) by one member of the research group not involved with the original intervention studies (TO). We then aimed to select two normal weight women from the same study period for each overweight woman by identifying women with their pre-pregnancy BMI representing the mean of the total population of normal weight women in the corresponding study period. On this basis, a total of 125 pregnant women were included in the present study, of whom 41 were overweight and 83 normal weight (Table 1). One woman pregnant in 2017 was included in the microbiota analyses but excluded from those pertaining to BMI as the pre-pregnancy BMI could not be confirmed.

### Ethics

The study was conducted in accordance with the Helsinki Declaration of 1975 and other relevant ethics guidelines and regulations. The original clinical studies were approved by the Ethics committee of the Hospital District of Southwest Finland. Oral and written informed consent was obtained from all subjects.

### DNA extraction and 16S rRNA amplicon sequencing

Total DNA was extracted from the fecal material (approx. 100 mg) using the automated assisted method based on magnetic beads (Maxwell® RSC Instrument coupled with Maxwell RSC Pure Food GMO and authentication kit, Promega, Spain) following the manufacturer’s instructions with previous treatments to improve the DNA extraction. In brief, samples were treated with lysozyme (20 mg/mL) and mutanolysin (5 U/mL) for 60 min at 37°C and a preliminary step of cell disruption with 3-μm diameter glass beads during 1 min at 6 m/s by a bead beater FastPrep 24-5 G Homogenizer (MP Biomedicals). Purification of the DNA was performed using DNA Purification Kit (Macherey-Nagel, Duren, Germany) according to manufacturer’s instructions. DNA concentration was measured using Qubit® 2·0 Fluorometer (Life Technology, Carlsbad, CA, USA) for further analysis.

DNA libraries were constructed with the amplification of the V3-V4 variable region of the 16S rRNA gene as described previously.^32^ A multiplexing step was conducted by the NextEra XT Index Kit (FC-131-2001) (Illumina, San Diego, CA, USA) and DNA quality of the library PCR product was measured by a Bioanalyzer DNA 1000 chip (Agilent Technologies, Santa Clara, CA, USA) to verify the size; the expected size on a Bioanalyzer trace is ~550 bp. The libraries were sequenced using was a 2 × 300 bp paired-end run on a Illumina platform (FISABIO sequencing service, Valencia, Spain) according to manufacturer instructions. Controls during DNA extraction and PCR amplification were also included and sequenced.

### Computational and statistical analysis

Residual adaptors were removed from the raw sequences using Trimmomatic software.^33^ A DADA2 pipeline was used to achieve quality filtering, sequence joining, and chimera removal.^34^ Taxonomic assignment was conducted using the Silva v132 database with the addition of the species level classification by the same database.^35^ Samples with less than 1000 reads were removed from the study. Resulted taxonomical tables were processed using phyloseq package^36^ for further statistical analysis in Rstudio environment.^37^ Alpha diversity indexes were calculated using the mentioned package after rarefaction to the minimum reads (1052). Differences in alpha diversity index were assessed by Kruskal–Wallis test followed by post-hoc Dunn’s test with Benjamini–Hochberg correction for p-value adjustment. Beta diversity was studied based on the Bray-Curtis distance and permutational multivariate analysis of variance (Adonis test) was assessed to determine differences in the overall microbial community structure. These were also represented in canonical correspondence analysis (CCA) using several R packages including ggplots,^38^ ggordiplot,^39^ FactoMiner,^40^ and FactoExtra.^41^

Differential abundance of taxa was tested using Kruskal–Wallis test after CLR normalization with a false discovery rate (FDR) adjustment for multiple comparisons (referred as q in the text). A p-value <.05 and q < 0.2 were considered the statistical thresholds for significance. Linear discriminant analysis (LDA) effect sized (LEfSe) analysis^42^ was performed for the biomarker discovery using a size-effect cutoff of 2.0 on the logarithmic LDA score. All differential abundance test including Lefse analysis were performed using their specific functions form the MicrobiomeMarker package.^43^ To assess the potential impact of BMI on the pregnant microbiota, general lineal models were performed using Maaslin2 algorithm.^44^ The 16S rRNA gene sequence data generated is available through NCBI Sequence Read Archive Database under project accession number BioProject ID PRJNA844901.

### Short Chain Fatty Acid (SCFA) profiles

The short chain fatty acid (SCFA) profiles were measured on fecal supernatant by GC- MS according to Eberhart et al.^45^ by use of a gas chromatography system Agilent GC 7890B − 5977B (Agilent Technologies, Palo Alto, CA, USA) fitted with a column DB- FATWAX, (30 m, 0 × 0.25 mm x 0.25 μm operated in split mode (20:1). The conditions were as follows: Oven temperature program: 100°C for 3 min, ramped to 100°C at a rate of 5°C min-1, to 150°C for 1 min, to 200°C at a rate of 20°C min-1, and finally held at 200 for 5 min. Helium was used as a carrier gas at a flow rate of 1 mL min-1; inlet temperature of 250°C.

Differences in the concentration of each SCFA according to the time period were determined by Kruskal–Wallis test. A CCA was also assessed to study the associations between the production of each individual SCFA with the general pattern of the specific decade. Specific Spearman’s rank correlations between microbial genera and SCFA were obtained and built by use of gplots package^46^ after normalization of the microbiota data as relative abundance using the microbiome package.^47^

## Supplementary Material

Supplemental Material

## Data Availability

The 16S rRNA gene sequence data generated is available through NCBI Sequence Read Archive Database under project accession number BioProject ID PRJNA844901 is available from National Center for Biotechnology Information (NCBI) repository (https://www.ncbi.nlm.nih.gov/bioproject/PRJNA844901). All supporting data are included in the manuscript.

## Abbreviations

BMI: Body Mass Index
FDR: False Discovery Rate
CCA: Canonical Correspondence Analysis
GC-MS: Gas Chromatography-Mass Spectrometry
LDA: Linear Discriminant Analysis
PERMANOVA: Permutational Multivariate Analysis Of Variance
SCFAs: Short Chain Fatty Acids

## References

[1] Bach JF. The effect of infections on susceptibility to autoimmune and allergic diseases. N Engl J Med. 2002;347(12):911–12. doi:10.1056/NEJMra020100. 12239261

[2] NCD Risk Factor Collaboration (NCD-RisC). Trends in adult body-mass index in 200 countries from 1975 to 2014: a pooled analysis of 1698 population-based measurement studies with 19·2 million participants. Lancet. 2016;387(10026):1377–1396. doi:10.1016/S0140-6736(16)30054-X. 27115820 PMC7615134

[3] Aris IM, Block JP. Childhood obesity interventions—going beyond the individual. JAMA Pediatr. 2022;176(1):e214388. doi:10.1001/jamapediatrics.2021.4388. 34747988

[4] Wankhade UD, Thakali KM, Shankar K. Persistent influence of maternal obesity on offspring health: Mechanisms from animal models and clinical studies. Mol Cell Endocrinol. 2016;435:7–19. doi:10.1016/j.mce.2016.07.001. 27392497

[5] Heslehurst N, Vieira R, Akhter Z, Bailey H, Slack E, Ngongalah L, Pemu A, Rankin J, Persson LÅ. The association between maternal body mass index and child obesity: A systematic review and meta-analysis. PLoS Med. 2019;16(6):e1002817. doi:10.1371/journal.pmed.1002817. 31185012 PMC6559702

[6] Liem ET, van Buuren S, Sauer PJJ, Jaspers M, Stolk RP, Reijneveld SA. Growth during infancy and childhood, and adiposity at age 16 years: ages 2 to 7 years are pivotal. J Pediatr. 2013;162(2):287–292. doi:10.1016/j.jpeds.2012.07.053. 22985721

[7] Dominguez-Bello MG, Godoy-Vitorino F, Knight R, Blaser M. Role of the microbiome in human development. Gut. 2019;68(6):1108–1114. doi:10.1136/gutjnl-2018-317503. 30670574 PMC6580755

[8] Rautava S, Luoto R, Salminen S, Isolauri E. Microbial contact during pregnancy, intestinal colonization and human disease. Nat Rev Gastroenterol Hepatol. 2012;9(10):565–576. doi:10.1038/nrgastro.2012.144. 22890113

[9] Tremaroli V, Bäckhed F. Functional interactions between the gut microbiota and host metabolism. Nature. 2012;489(7415):242–249. doi:10.1038/nature11552. 22972297

[10] Bäckhed F, Ding H, Wang T, Hooper LV, Koh GY, Nagy A, Semenkovich CF, Gordon JI. The gut microbiota as an environmental factor that regulates fat storage. Proc Natl Acad Sci USA. 2004;101(44):15718–15723. doi:10.1073/pnas.0407076101. 15505215 PMC524219

[11] Cani PD, Amar J, Iglesias MA, Poggi M, Knauf C, Bastelica D, Neyrinck AM, Fava F, Tuohy KM, Chabo C, et al. Metabolic endotoxemia initiates obesity and insulin resistance. Diabetes. 2007;56(7):1761–1772. doi:10.2337/db06-1491. 17456850

[12] Blaser MJ, Falkow S. What are the consequences of the disappearing human microbiota? Nat Rev Microbiol. 2009;7(12):887–894. doi:10.1038/nrmicro2245. 19898491 PMC9354563

[13] Finlay BB, Amato KR, Azad M, Blaser MJ, Bosch TCG, Chu H, Dominguez- Bello MG, Ehrlich SD, Elinav E, Geva-Zatorsky N, et al. The hygiene hypothesis, the COVID pandemic, and consequences for the human microbiome. Proc Natl Acad Sci USA. 2021;118(6):e2010217118. doi:10.1073/pnas.2010217118. 33472859 PMC8017729

[14] Śliżewska K, Włodarczyk M, Sobczak M, Barczyńska R, Kapuśniak J, Socha P, Wierzbicka-Rucińska A, Kotowska A. Comparison of the activity of fecal enzymes and concentration of SCFA in healthy and overweight children. Nutrients. 2023;15(4):987. doi:10.3390/nu15040987. 36839343 PMC9966664

[15] Subramanian S, Blanton LV, Frese SA, Charbonneau M, Mills DA, Gordon JI. Cultivating healthy growth and nutrition through the gut microbiota. Cell. 2015;161(1):36–48. doi:10.1016/j.cell.2015.03.013. 25815983 PMC4440586

[16] Zhao L. The gut microbiota and obesity: from correlation to causality. Nat Rev Microbiol. 2013;11(9):639–647. doi:10.1038/nrmicro3089. 23912213

[17] Tasnim N, Abulizi N, Pither J, Hart MM, Gibson DL. Linking the gut microbial ecosystem with the environment: does gut health depend on where we live? Front Microbiol. 2017;8:1935. doi:10.3389/fmicb.2017.01935. 29056933 PMC5635058

[18] Rothschild D, Weissbrod O, Barkan E, Kurilshikov A, Korem T, Zeevi D, Costea PI, Godneva A, Kalka IN, Bar N, et al. Environment dominates over host genetics in shaping human gut microbiota. Nature. 2018;555(7695):210–215. doi:10.1038/nature25973. 29489753

[19] Uzan-Yulzari A, Turta O, Belogolovski A, Ziv O, Kunz C, Perschbacher S, Neuman H, Pasolli E, Oz A, Ben-Amram H, et al. Neonatal antibiotic exposure impairs child growth during the first six years of life by perturbing intestinal microbial colonization. Nat Commun. 2021;12(1):443. doi:10.1038/s41467-020-20495-4. 33500411 PMC7838415

[20] De Filippo C, Di Paola M, Ramazzotti M, Albanese D, Pieraccini G, Banci E, Miglietta F, Cavalieri D, Lionetti P. Diet, environments, and gut microbiota. A preliminary investigation in children living in rural and urban Burkina Faso and Italy. Front Microbiol. 2017;8:1979. doi:10.3389/fmicb.2017.01979. 29081768 PMC5645538

[21] Kaartinen NE, Tapanainen H, Männistö S, Reinivuo H, Virtanen SM, Jousilahti P, Koskinen S, Valsta LM. Changes in food consumption and nutrient intake in Finnish adults 1997–2017: A FinDiet survey. Suomen Lääkärilehti. 2021;76:273–280. (Finnish).

[22] Hoppu U, Kalliomäki M, Isolauri E. Maternal diet rich in saturated fat during breastfeeding is associated with atopic sensitization of the infant. Eur J Clin Nutr. 2000;54(9):702–705. doi:10.1038/sj.ejcn.1601079. 11002382

[23] Piirainen T, Isolauri E, Lagström H, Laitinen K. Impact of dietary counselling on nutrient intake during pregnancy: a prospective cohort study. Br J Nutr. 2006;96(6):1095–1104. doi:10.1017/BJN20061952. 17181885

[24] Kinnunen TI, Puhkala J, Raitanen J, Ahonen S, Aittasalo M, Virtanen SM, Luoto R. Effects of dietary counselling on food habits and dietary intake of Finnish pregnant women at increased risk for gestational diabetes - a secondary analysis of a cluster-randomized controlled trial. Matern Child Nutr. 2014;10(2):184–197. doi:10.1111/j.1740-8709.2012.00426.x. 22735030 PMC6860280

[25] Koren O, Goodrich JK, Cullender TC, Spor A, Laitinen K, Bäckhed HK, Gonzalez A, Werner JJ, Angenent LT, Knight R, et al. Host remodeling of the gut microbiome and metabolic changes during pregnancy. Cell. 2012;150(3):470–480. doi:10.1016/j.cell.2012.07.008. 22863002 PMC3505857

[26] Dreisbach C, Alhusen J, Prescott S, Dudley D, Trinchieri G, Siega-Riz AM. Metagenomic characterization of the maternal prenatal gastrointestinal microbiome by pregravid BMI. Obesity (Silver Spring, Md). 2023;31:412–422. doi:10.1002/oby.23659. 36562201 PMC10108029

[27] Pinto Y, Frishman S, Turjeman S, Eshel A, Nuriel-Ohayon M, Shrossel O, Ziv O, Walters W, Parsonnet J, Ley C, et al. Gestational diabetes is driven by microbiota-induced inflammation months before diagnosis. Gut. 2023;72(5):918–928. doi:10.1136/gutjnl-2022-328406. 36627187 PMC10086485

[28] Renz H, Skevaki C. Early life microbial exposures and allergy risks: opportunities for prevention. Nat Rev Immunol. 2021;21(3):177–191. doi:10.1038/s41577-020-00420-y. 32918062

[29] Wirth U, Garzetti D, Jochum LM, Spriewald S, Kühn F, Ilmer M, Lee SML, Niess H, Bazhin AV, Andrassy J, et al. Microbiome analysis from paired mucosal and fecal samples of a colorectal cancer biobank. Cancers Basel. 2020;12(12):3702. doi:10.3390/cancers12123702. 33317136 PMC7762977

[30] Kalliomäki M, Salminen S, Arvilommi H, Kero P, Koskinen P, Isolauri E. Probiotics in primary prevention of atopic disease: a randomised placebo- controlled trial. Lancet. 2001;357(9262):1076–1079. doi:10.1016/S0140-6736(00)04259-8. 11297958

[31] Rautava S, Kainonen E, Salminen S, Isolauri E. Maternal probiotic supplementation during pregnancy and breast-feeding reduces the risk of eczema in the infant. J Allergy Clin Immunol. 2012;130(6):1355–1360. doi:10.1016/j.jaci.2012.09.003. 23083673

[32] García-Mantrana I, Selma-Royo M, González S, Parra-Llorca A, Martínez-Costa C, Collado MC. Distinct maternal microbiota clusters are associated with diet during pregnancy: impact on neonatal microbiota and infant growth during the first 18 months of life. Gut Microbes. 2020;11(4):962–978. doi:10.1080/19490976.2020.1730294. 32167021 PMC7524361

[33] Bolger AM, Lohse M, Usadel B. Trimmomatic: a flexible trimmer for Illumina sequence data. Bioinformatics. 2014;30(15):2114–2120. doi:10.1093/bioinformatics/btu170. 24695404 PMC4103590

[34] Callahan BJ, McMurdie PJ, Rosen MJ, Han AW, Johnson AJ, Holmes SP. DADA2: high-resolution sample inference from Illumina amplicon data. Nat Methods. 2016;13(7):581–583. doi:10.1038/nmeth.3869. 27214047 PMC4927377

[35] Quast C, Pruesse E, Yilmaz P, Gerken J, Schweer T, Yarza P, Peplies J, Glöckner FO. The SILVA ribosomal RNA gene database project: improved data processing and web-based tools. Nucleic Acids Res. 2013;41(Database issue):D590–596. doi:10.1093/nar/gks1219. 23193283 PMC3531112

[36] McMurdie PJ, Holmes S, Watson M. Phyloseq: an R package for reproducible interactive analysis and graphics of microbiome census data. PLoS One. 2013;8(4):e61217. doi:10.1371/journal.pone.0061217. 23630581 PMC3632530

[37] RStudio | Open source & professional software for data science teams [Internet]. [accessed 2022 Mar 22]. https://www.rstudio.com/

[38] Wickham H. ggplot2 [Internet]. Cham: Springer International Publishing; 2016 [accessed 2022 Mar 22]. (Use R!). http://link.springer.com/10.1007/978-3-319-24277-4

[39] jfq3. ggordiplots [Internet]. 2021 [accessed 2022 Mar 22]. https://github.com/jfq3/ggordiplots

[40] Lê S, Josse J, Husson F. FactoMineR: an R package for multivariate analysis. J Stat Soft. 2008;25(1):1–18. doi:10.18637/jss.v025.i01.

[41] Kassambara A, Mundt F Factoextra: extract and visualize the results of multivariate data analyses [Internet]. 2020 [2022 Mar 22]. https://CRAN.R-project.org/package=factoextra

[42] Segata N, Izard J, Waldron L, Gevers D, Miropolsky L, Garrett WS, Huttenhower C. Metagenomic biomarker discovery and explanation. Genome Biol. 2011;12(6):R60. doi:10.1186/gb-2011-12-6-r60. 21702898 PMC3218848

[43] Microbiome biomarker analysis toolkit [Internet]. [accessed 2022 Mar 22]. https://yiluheihei.github.io/microbiomeMarker/

[44] Mallick H, Rahnavard A, McIver LJ, Ma S, Zhang Y, Nguyen LH, Tickle TL, Weingart G, Ren B, Schwager EH, et al. Multivariable association discovery in population-scale meta-omics studies. PLoS Comput Biol. 2021;17(11):e1009442. doi:10.1371/journal.pcbi.1009442. 34784344 PMC8714082

[45] Eberhart BL 2nd, Wilson AS, O’Keefe SJD, Ramaboli MC, Nesengani LT. A simplified method for the quantitation of short-chain fatty acids in human stool. Anal Biochem. 2021;612:114016. doi:10.1016/j.ab.2020.114016. 33188741

[46] Gplots: various R programming tools for plotting data – scienceopen [Internet]. [accessed 2022 Mar 22]. https://www.scienceopen.com/document?vid=0e5d8e31-1fe4-492f-a3d8-8cd71b2b8ad9

[47] Leo Lahti SS Tools for microbiome analysis in R. Version. 2017. [Internet] http://microbiome.github.com/microbiome.

