# Supplementary material for: Shifting pattern of gut microbiota in pregnant women two decades apart – an observational study
Source: Gut Microbes. 2023 Jul 19;15(1):2234656. doi: 10.1080/19490976.2023.2234656 (PMC10361139; doi:10.1080/19490976.2023.2234656)

**Supplementary Table S1.** Results from multivariate analysis considering time period (1997, 2007 or 2017) and maternal overweight (body mass index greater or equal to 25 kg/m^2)^.

|  | **Maaslin Coefficient** | **Standard error** | **p-value** | **q-value** |
| --- | --- | --- | --- | --- |
| **Time period (Reference: 1997)** | | | | |
| **2007** | | | | |
| *Streptococcus* | 1.75 | 0.61 | 0.005 | 0.109 |
| *Fournierella* | -0.94 | 0.31 | 0.003 | 0.109 |
| *Lachnospiraceae NC2004_group* | -1.17 | 0.41 | 0.005 | 0.109 |
| *Hydrogenoanaerobacterium* | -1.17 | 0.4 | 0.004 | 0.109 |
| *Phocea* | -1.17 | 0.29 | <0.001 | 0.011 |
| *Faecalibacterium* | -1.43 | 0.52 | 0.007 | 0.131 |
| *Lachnospiraceae UCG-008* | -1.57 | 0.43 | <0.001 | 0.026 |
| *Lachnospiraceae NK4A136_group* | -1.6 | 0.56 | 0.005 | 0.109 |
| *Bilophila* | -1.68 | 0.6 | 0.006 | 0.131 |
| *Anaerofilum* | -1.8 | 0.45 | <0.001 | 0.011 |
| *Ruminococcaceae_UCG-003* | -2.06 | 0.63 | 0.001 | 0.065 |
| *Lachnospira* | -2.44 | 0.61 | <0.001 | 0.011 |
| *Lachnospiraceae_UCG-004* | -2.51 | 0.63 | <0.001 | 0.011 |
| **2017** | | | | |
| *Lactococcus* | 2.81 | 0.92 | 0.003 | 0.109 |
| *Fournierella* | -1.26 | 0.38 | 0.001 | 0.065 |
| *Lachnospiraceae_NC2004_group* | -1.56 | 0.5 | 0.002 | 0.1 |
| *Hydrogenoanaerobacterium* | -1.7 | 0.5 | 0.001 | 0.055 |
| *GCA-900066225* | -1.76 | 0.6 | 0.004 | 0.109 |
| *Leuconostoc* | -1.87 | 0.64 | 0.004 | 0.109 |
| *Lachnospira* | -2.02 | 0.76 | 0.008 | 0.156 |
| *Marvinbryantia* | -2.21 | 0.76 | 0.004 | 0.109 |
| *Pseudoflavonifractor* | -2.28 | 0.77 | 0.004 | 0.109 |
| *Coprococcus_1* | -2.6 | 0.8 | 0.001 | 0.065 |
| *Anaerofilum* | -2.79 | 0.56 | <0.001 | 0.001 |
| **Pre-pregnancy overweight (Reference: normal weight)** | | | | |
| **Overweight** | | | | |
| *Bacteroides* | -0.89 | 0.33 | 0.008 | 0.146 |

**Supplementary Figure S1. Participant flow chart for the pregnant women at each time period.** The subjects were selected from the original study population based on availability of fecal samples and data on body mass index (BMI). Women with pre- pregnancy BMI ≥ 25 kg/m^2^ were considered overweight. Subjects representing the population mean were then chosen as normal weight control subjects.


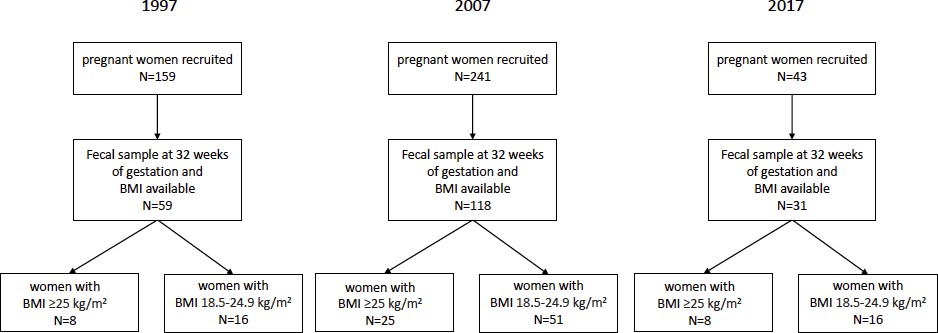


**Supplementary Figure S2. Effect of the studied variables in pregnant women microbiota.** a) Factors affecting gut microbiota assessed by Adonis test on the Bray- Curtis distance. * p<0,05 (The ADONIS general model with all factors F= 1.12, R2=0.05 and p=0.085). b) Boxplots showing the relative abundance of genera with significant differences between time periods. Statistical analysis was performed using Kruskal-Wallis test on cantered log ratio (CLR) normalized data. Those genera that showed a difference with a q<0.2 were plotted.

**a)**


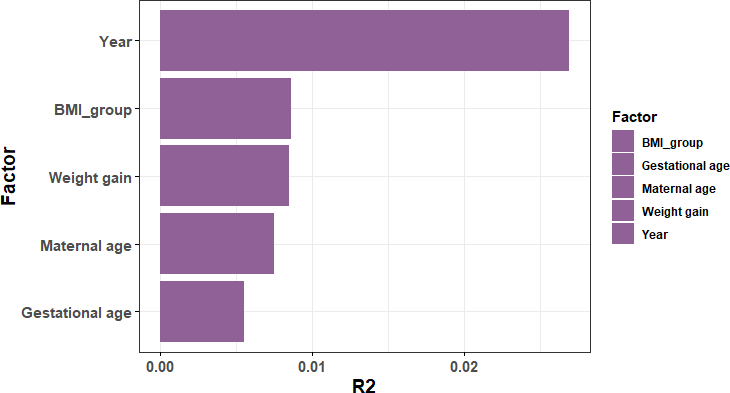


**b)**


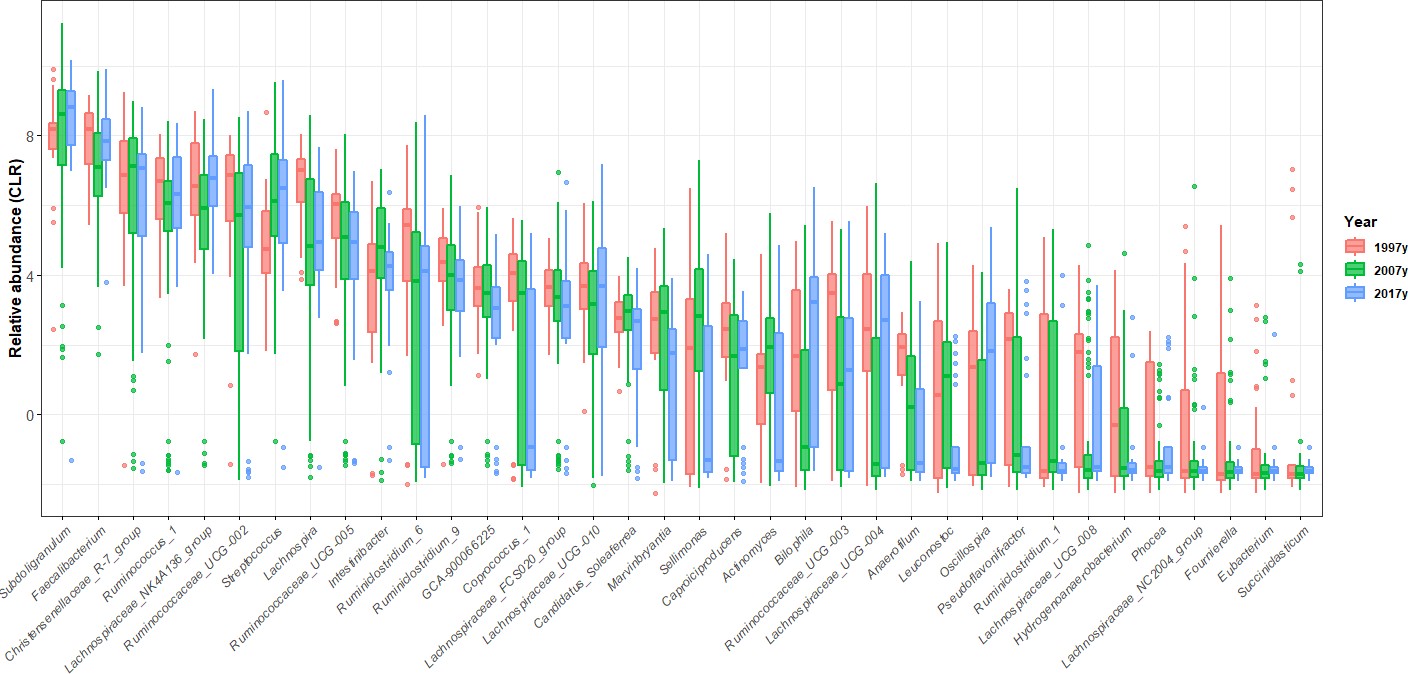


**Supplementary Figure S3. Associations between fecal SCFA quantification and gut microbiota in pregnant women.** Heatmap plot showing the correlation between gut microbiota at genus level and the quantification of the short chain fatty acid (SCFA) in the fecal samples. Microbiota data were normalized before correlation analysis and only those genera that showed at least one significant relation were included in the final plot. AA, Acetic acid; PA, propionic acid; IBA, isobutyric acid; CA, caproic acid; BA, butyric acid; IVA, Isovaleric acid; VA, valeric acid, Total, total SCFA quantification.


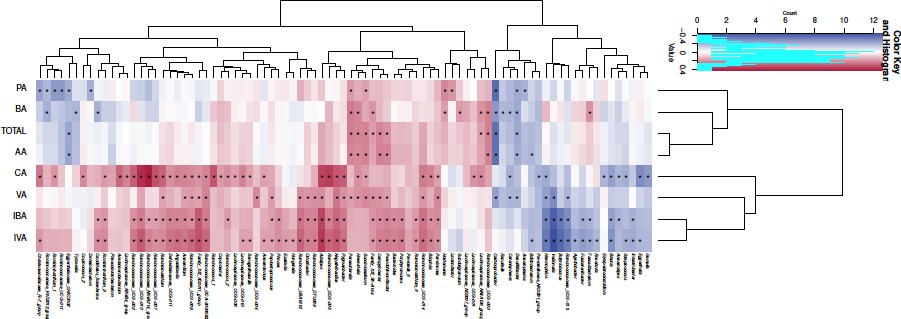

Supplement: Supplemental Material [file KGMI_A_2234656_SM9982.docx]
